# Supplementary material for: Triple-chord trussed submerged floating tunnels: hybrid construction concept, feasibility and design
Source: Commun Eng. 2025 Jul 1;4:117. doi: 10.1038/s44172-025-00454-x (PMC12217642; doi:10.1038/s44172-025-00454-x)
Supplement: Supplementary file 2 — Supplementary Information [file 44172_2025_454_MOESM2_ESM.docx]

**Triple-chord trussed submerged floating tunnels: hybrid construction concept, feasibility and design**

Fa-Cheng Wang^1,2^*, Tao Zhuge^1^, Zheng-Qing Cheng^1,3^, Lin-Hai Han^1^, Jian-Min Zhang^1,2^, Leroy Gardner ^4,1^ *

^1^ School of Civil Engineering, Tsinghua University, Beijing 100084, PR China

^2^ Institute for Ocean Engineering, Tsinghua University, Beijing 100084, PR China

^3^ Railway Engineering Research Institute, China Academy of Railway Sciences Corporation Limited, Beijing 100081, PR China

^4^ Department of Civil and Environmental Engineering, Imperial College London, SW7 2AZ, United Kingdom

**Contents**

**Supplementary Table 1 - 3**

**Supplementary Data 1 - 2**

**Supplementary Table 1. Simulation results of maximum lateral displacement and stiffness of SFT schemes**

| Scheme | *u*_m_ (mm) | *R* (kN) | *K*_s_ (kN/mm) |
| --- | --- | --- | --- |
| Scheme A | 228.9 | 19976 | 87.2 |
| Scheme B | 137.3 | 44001 | 321.2 |
| Scheme C | 717.6 | 43171 | 60.1 |
| Scheme D | 285.2 | 41512 | 145.6 |

*u*_m_ is the maximum lateral displacement of the scheme; *R* is the lateral reaction force at the supporting points; *K*_s_ is the structure stiffness.

**Supplementary Table 2 | Comparison of ultimate moments**

| Type | Specimen | *f*_yo_  (MPa) | *f*_yi_  (MPa) | *f*_cu_  (MPa) | *M*_u_  (kN·m) | *M*_Predict_  (kN·m) | *M*_Predict_/*M*_u_ |
| --- | --- | --- | --- | --- | --- | --- | --- |
| CFDST beam | B1-1 | 618 | 356 | 46.5 | 605 | 582 | 0.962 |
|  | B1-2 | 618 | 356 | 46.5 | 575 | 582 | 1.012 |
|  | B2-1 | 618 | 357 | 46.5 | 589 | 574 | 0.974 |
|  | B2-2 | 618 | 357 | 46.5 | 577 | 574 | 0.995 |
| CFST truss | T8-1 | 324 (top chord)  316 (bottom chord) | - | 69.9 | 202 | 215 | 1.064 |
|  | T8-2 |  |  |  | 207 | 215 | 1.039 |
|  |  |  |  |  |  | Mean | 1.008 |
|  |  |  |  |  |  | COV | 0.035 |

*f*_yo_, *f*_yi_, and *f*_cu_ denote the yield strength of the outer steel tube, inner steel tube and concrete cubic strength, respectively. *M*_u_ denotes the ultimate moment obtained from experiments; *M*_predict_ denotes the ultimate moment predicted by the numerical models.

**Supplementary Table 3 | Simulation results for 3-D FE and simplified fibre models**

| Model name | *L*_s_ (m) | *λ* | *u*_m,3-D FE_ (mm) | *u*_m,simplified_ (mm) | *Δ* |
| --- | --- | --- | --- | --- | --- |
| L-1 | 4.8 | 25.3 | 34.0 | 43.5 | 0.279 |
| L-2 | 7.2 | 38.0 | 101.3 | 109.5 | 0.081 |
| L-3 | 9.6 | 50.7 | 227.9 | 234.8 | 0.030 |
| L-4 | 12.0 | 63.4 | 435.4 | 438.7 | 0.008 |
| L-5 | 14.4 | 76.0 | 740.2 | 738.9 | 0.002 |

*L*_s_ denotes the length of the structure. *λ* denotes the slenderness ratio of the structure. *u*_m,3-D FE_ and *u*_m,simplified_ denote the maximum displacement prediction given by three-dimensional solid finite element (3-D FE) models and simplified fibre models, respectively. *Δ* denotes the relative error of the simplified model predictions, calculated by |*u*_m,simplified_ - *u*_m,3-D FE_| / *u*_m,3-D FE_.

**Supplementary Data 1 and 2 are given in corresponding excel files.**
